# The Electrochemical and Mechanical Behavior of Bulk and Porous Superelastic Ti‒Zr-Based Alloys for Biomedical Applications

**DOI:** 10.3390/ma12152395

**Published:** 2019-07-27

**Authors:** Yulia Zhukova, Anastasia Korobkova, Sergey Dubinskiy, Yury Pustov, Anton Konopatsky, Dmitry Podgorny, Mikhail Filonov, Sergey Prokoshkin, Vladimir Brailovski

**Affiliations:** 1 Center of Nanomaterials and Nanotechnologies, National University of Science and Technology “MISIS”, 4 Leninskiy prospekt, Moscow 119049, Russia; 2 Metal Forming Department, National University of Science and Technology “MISIS”, 4 Leninskiy prospekt, Moscow 119049, Russia; 3 Materials Science of Semiconductors and Dielectrics Department, National University of Science and Technology “MISIS”, 4 Leninskiy prospekt, Moscow 119049, Russia; 4 Department of Functional Nanosystems and High-Temperature Materials, National University of Science and Technology “MISIS”, 4 Leninskiy prospekt, Moscow 119049, Russia; 5 Shape Memory Alloys and Intelligent Systems Laboratory (LAMSI), École de Technologie Supérieure (ETS), 1100 Notre-Dame Street West, Montreal, QC H3C 1K3, Canada

**Keywords:** shape memory materials, superelastic materials, implants, thermomechanical treatment, microstructure, martensitic transformation, corrosion resistance, oxide film, biocompatibility

## Abstract

Titanium alloys are well recognized as appropriate materials for biomedical implants. These devices are designed to operate in quite aggressive human body media, so it is important to study the corrosion and electrochemical behavior of the novel materials alongside the underlying chemical and structural features. In the present study, the prospective Ti‒Zr-based superelastic alloys (Ti-18Zr-14Nb, Ti-18Zr-15Nb, Ti-18Zr-13Nb-1Ta, atom %) were analyzed in terms of their phase composition, functional mechanical properties, the composition and structure of surface oxide films, and the corresponding corrosion and electrochemical behavior in Hanks’ simulated biological solution. The electrochemical parameters of the Ti-18Zr-14Nb material in bulk and foam states were also compared. The results show a significant difference in the functional performance of the studied materials, with different composition and structure states. In particular, the positive effect of the thermomechanical treatment regime, leading to the formation of a favorable microstructure on the corrosion resistance, has been revealed. In general, the Ti-18Zr-15Nb alloy exhibits the optimum combination of functional characteristics in Hanks’ solution, while the Ti-18Zr-13Nb-1Ta alloy shows the highest resistance to the corrosion environment. The Ti-18Zr-14Nb-based foam material exhibits slightly lower passivation kinetics as compared to its bulk equivalent.

## 1. Introduction

Since the 1950s, titanium and its alloys have been widely used in medicine as materials for orthopedic implants due to the renowned combination of high mechanical properties, relatively low stiffness and density, nonmagnetic characteristics, which are essential for magnetic resonance imaging, and pronounced biocompatibility, which allows for maintaining a direct bond with bone [1,2,3,4]. However, the issues of low wear resistance, insufficient bioactivity, and the mechanical behavior mismatch between the implant and the surrounding bone tissue, leading to stress shielding, are still problems to be solved [3,5,6,7,8]. The latter difference is underlined by the fact that natural bone possesses a low Young’s modulus and hysteresic mechanical behavior upon loading‒unloading cycles. From this point of view, biomechanically compatible titanium implant materials can be designed in two different ways [9,10,11].

The first approach to create biomechanically compatible metallic materials includes the development of new alloys with a Young’s modulus approaching that of bone (for example, [12,13,14,15,16,17,18]). Superelastic mechanical behavior is another significant effect contributing to the biomechanical compatibility, as it resembles the loading‒unloading behavior of bone tissue [19]. The superelasticity effect can be exhibited by a material due to reversible martensitic transformation upon loading and unloading, resulting in reversible strain values of several percentage points [20]. Moreover, it is established that the presence of this phase transition is accompanied by lattice softening, leading to a significant decrease in the Young’s modulus [21,22]. In this context, near-equiatomic Ti‒Ni alloys represent the most widely used shape memory and superelastic materials for various technical and biomedical applications [23]. However, their use as an intraosseous implant material is limited by the nickel toxicity. Therefore, Ni-free superelastic titanium alloys have been designed and characterized extensively over the past two decades [24,25,26].

The second approach to creating biomechanically compatible metallic implants involves the production of porous metal structures using powder metallurgy, electrochemical synthesis, or additive manufacturing techniques [9,10,26,27,28,29,30,31,32,33]. It has been shown that the Young’s modulus of the produced porous structures can be adjusted to match that of the softest bone tissues [9,10,26].

Due to the fact that load-bearing intraosseous implants are designed to operate in quite aggressive body media, it is of great practical importance to study the corrosion and electrochemical behavior of these prospective materials, including tests in simulated physiological conditions [34]. The chemical composition and thickness of the passive oxide layer formed on the material surface also play an important role in the implant’s biocompatibility and should be studied carefully [35,36].

Despite the high corrosion resistance, titanium and its alloys can undergo certain types of corrosion in the human body [37,38,39,40,41,42]: general corrosion, localized corrosion (pitting and crevice types), medium-assisted degradation (corrosion cracking, corrosion fatigue, hydrogen embrittlement), or tribocorrosion (wear-accelerated corrosion).

Despite the significant number of publications (including the abovementioned ones) addressing different aspects of the Ni-free Ti-based alloys’ development, works focusing on practically relevant combinations of functional properties, i.e., the biomechanical behavior and corrosion resistance in terms of the underlying microstructural features, are still lacking.

The present paper aims at a comparative study of the selected prospective Ni-free superelastic alloys (Ti-18Zr-14Nb, Ti-18Zr-15Nb, Ti-18Zr-13Nb-1Ta, atom %) with a special focus on their corrosion and electrochemical characterization in Hanks’ physiological solution, including that under cycling loading conditions. The results are discussed in correlation with the alloy’s phase composition, mechanical behavior, and surface oxide film composition after exposure to Hanks’ solution. The electrochemical characteristics of the bulk and Ti-18Zr-14Nb-based foam material samples are compared as well.

## 2. Materials and Methods 

The three compositions of prospective Ti‒Zr-based alloys listed in Table 1 were chosen for the present study on the basis of the results published in [12,14].

For this study, ingots weighing 40 g (and approximately 10 × 10 × 70 mm in size) were produced by vacuum arc remelting with a non-consumable tungsten electrode under an argon protective atmosphere. For each ingot, the remelting process was repeated five times to reach chemical and structural homogeneity, and the ingots were turned upside down after each remelting. The preparatory melting of pure titanium provided a low amount of impurities (O < 0.06, C < 0.04, N < 0.0008, H < 0.010, wt %). Crystallization was carried out in a water-cooled copper mold. The ingots were subsequently homogenized in an argon atmosphere at 1173 K for 3.6 ks to modify the as-cast structure.

Each ingot was sliced into 1.5-mm-thick plates using electric discharge machining. Afterward, all plates were subjected to thermomechanical treatment (TMT), which included cold rolling with a true logarithmic strain of *e* = 0.3 and post-deformation annealing at 873 K for 1.8 ks, followed by water quenching. The post-deformation annealing creates a nanosubgrained structure in β-phase of the studied alloys [14], which provides the best combination of functional properties of analogous alloys [43].

A Ti-18Zr-14Nb-based foam material with a porosity value of 50% was produced by a space-holder technique, as described in [27]. Metallic powder (particles of at most 50 μm in diameter) and polymethylmethacrylate powder (particles at most 250 μm in diameter) were mixed to achieve a homogeneous state in the proportion that ensured a porosity of 45–50%. The powder mixture was then compacted at a pressure of 200 MPa to form cylindrical rods 15 mm in diameter and up to 20 mm in height. The rods were subjected to pyrolysis in a vacuum furnace at 723 K for 7.2 ks, followed by sintering at 1673 K for 10.8 ks. Samples in the form of 3-mm-thick, 10-mm-diameter disks were spark-cut from the rods. Finally, the samples were washed in an ultrasonic bath for 300 s using acetone as the washing medium.

X-Ray diffraction analysis (XRD) was carried out using a PANalytical X’Pert PRO (Malvern Panalytical, Royston, UK) diffractometer operating with CuKα-radiation with an average X-ray wavelength of λ = 0.154178 nm. Rectangular (5 × 10 × 1 mm) thermomechanically treated samples were used for the XRD study. After mechanical polishing by abrasive paper, the hardened surface layer was removed by etching in 2HF:1HNO_3_:17H_2_O, 1HF:15HNO_3_:5H_2_O, 1HF:4HNO_3_:5H_2_O solutions. The X-ray diffractograms after TMT were recorded at room temperature (RT). For the final XRD recording, samples were subjected to additional plastic deformation (cold rolling) at RT with a residual strain of 5–7% in order to stabilize the α”-martensite and allow its XRD study at RT.

The lattice parameters of the studied phases were calculated from the angular coordinates of β and α” XRD peaks. The maximum β ⇄ α” transformation lattice strain, *ε_max_*, which is a crystallographic limit of the recovery strain, was calculated from the lattice parameters of the α”- and β-phases. The calculation procedure was based on the least-squares method and the extrapolation of the β-phase lattice parameters to θ = 90°, as described in [23,44].

The superelastic fatigue behavior of the samples subjected to TMT was studied using an MTS Mini Bionix (MTS Systems, Eden Prairie, MN, USA) testing machine. A series of 70 × 2 × 1 mm samples were prepared by electrical spark cutting. The functional long-term stability evaluation was carried out at RT during multi-cycle tensile testing with a maximum strain of 2% per cycle to sample failure.

The composition, chemical state, and element distribution profile near the alloy’s surface were studied by Auger electron spectroscopy (AES) using a PHI-680 Physical Electronics (Physical Electronics Inc., Chanhassen, MN, USA) Auger nanoprobe combined with Ar ion sputtering (estimated sputtering rate 8.3·10^−2^ nm/s [36]). Relative sensitivity factors for the differential mode elements were used to calculate the concentration.

Electrochemical measurements were carried out using an IPC Pro MF electronic potentiostat (Volta Co., Ltd., Saint-Petersburg, Russia) by the open circuit potential (OCP) and potentiodynamic voltammetry measurement methods (potential scan rate 0.2 mV/s) at (310 ± 1) K in Hanks’ simulated physiological solution containing (concentrations, g/L): 8 NaCl, 0.4 KCl, 0.04 Na_2_HPO_4_, 0.06 KH_2_PO_4_, 0.2 MgSO_4_·7H_2_O, 0.35 NaHCO_3_, 0.14 CaCl_2_, H_2_O (up to 1 dm^3^ of the solution), pH 7.4. Saturated silver chloride electrode (hereafter SSCE) was used as a reference electrode; a platinum electrode was used as an auxiliary electrode, with the immersed sample being a working electrode. Prior to each experiment, all samples were cleaned in distilled water for 600 s using a CT-405 (Wah Luen Technology Co., Ltd, Shantou, China) ultrasonic cleaner.

For electrochemical characterization under cyclic loading of superelastic Ti‒Zr-based alloys subjected to TMT, in comparison with Ti Grade 2, room-temperature fatigue testing was performed on samples of ~0.5 mm in thickness, ~3 mm in width, and ~120 mm in length on a specially designed pseudo-physiological test bench [45]. During each fatigue cycle, the strain (bending) amplitude was maintained at 1.5%; the cycling frequency was 0.9 Hz. The electrochemical behavior of the samples during cycling was monitored by OCP measurements using an IPC Pro MF (Volta Co., Ltd., Saint-Petersburg, Russia) potentiostat. The electrochemical measurements were carried out in both zero-loading and cyclic-loading states. At least five samples of each alloy were tested under the same conditions. Finally, Vega3 Tescan (Tescan, Brno, Czech Republic) scanning electron microscope (SEM) observations were carried out to analyze the different regions of fatigue fracture.

## 3. Results

### 3.1. XRD Study

**Figure 1:**
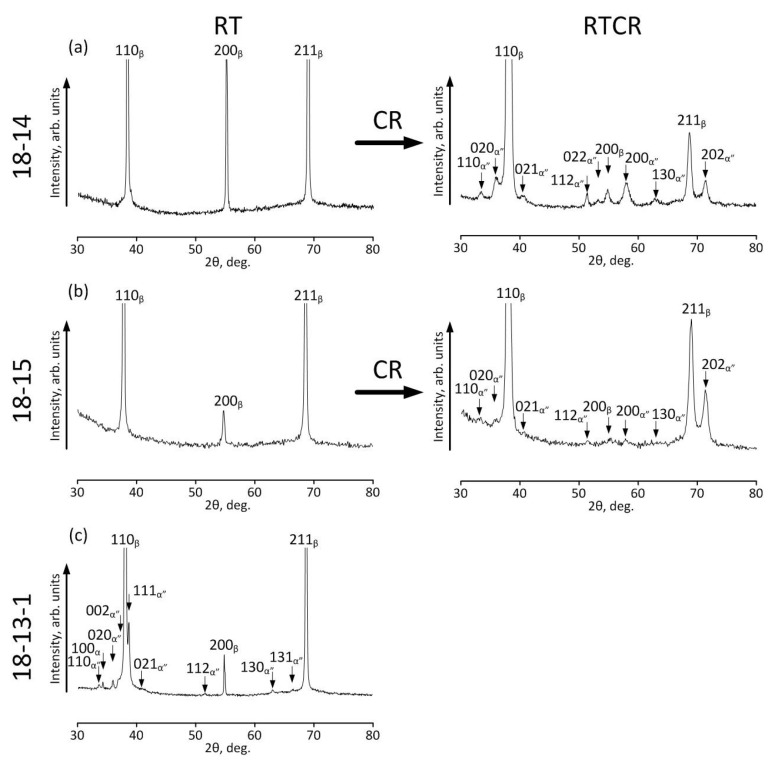
XRD profiles of (**a**) 18-14, (**b**) 18-15, and (**c**) 18-13-1 alloys after TMT, obtained at room temperature (RT) and after additional cold rolling deformation at room temperature (RTCR).

Figure 1 shows the XRD profiles of the 18-14, 18-15, and 18-13-1 alloys subjected to TMT. Initially, the XRD peaks obtained at room temperature (RT) of the 18-14 and 18-15 alloys correspond to the β-phase only, which is identified by its characteristic (110), (200), and (211) lines. Therefore, the samples of these compositions were additionally cold-rolled to 5–7% of residual strain in order to induce a certain quantity of stress-induced α”-martensite, as was done in [14]. This is necessary for calculating the crystallographic recovery strain limit of the alloys studied.

### 3.2. Functional Mechanical Behavior

**Figure 2:**
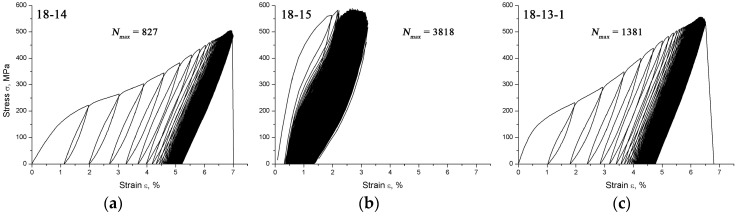
Typical sets of loading‒unloading diagrams obtained during multi-cycle testing of (**a**) 18-14, (**b**) 18-15, and (**c**) 18-13-1 alloys subjected to TMT.

Figure 2 shows typical loading-unloading diagrams obtained during multi-cycle testing of the 18-14, 18-15, and 18-13-1 alloys subjected to TMT.

It is evident from Figure 2 that the first cycles of all the samples feature imperfect superelastic behavior, with a residual strain of ~1.0% for the 18-14 and 18-13-1 alloys and ~0.5% for the 18-15 alloy. During the subsequent loading‒unloading cycling, the superelastic behavior becomes more pronounced, with this evolution being more evident for the 18-15 alloy. Table 2 lists the average number of cycles to failure (*N_max_*) for each alloy.

It can be seen that the highest *N_max_* value, which defines the functional fatigue life of the studied materials, is exhibited by the 18-15 alloy.

### 3.3. Surface Film Characterization

Table 3 and Table 4 show the results of an AES elemental analysis of the surface layers’ chemical compositions before and after exposure to Hanks’ solution for 604.8 ks. The compositions of the surface layers (up to 3 nm deep) were determined in the as-received state, while the compositions of the near-surface layers (about 100 nm deep) were obtained after ion sputtering. The contribution of the contaminating carbon (adsorbed hydrocarbon compounds) was not taken into account during the calculations for the other elements’ concentrations.

It is evident from Table 3 and Table 4 that before and after exposure to Hanks’ solution, the significant oxygen content is observed at the alloys’ surface, with its concentration rapidly decreasing with the increase in the analysis depth. The concentration values of the main alloying elements (Ti, Zr, Nb) are low in the near-surface layer, but increase after ion sputtering; Ta is not reliably identified in the 18-13-1 alloy.

The samples subjected to the Hanks’ solution show significant amounts of the solution’s components on the surface (about 40%), whose residual content after ion sputtering is about 10%.

**Figure 3:**
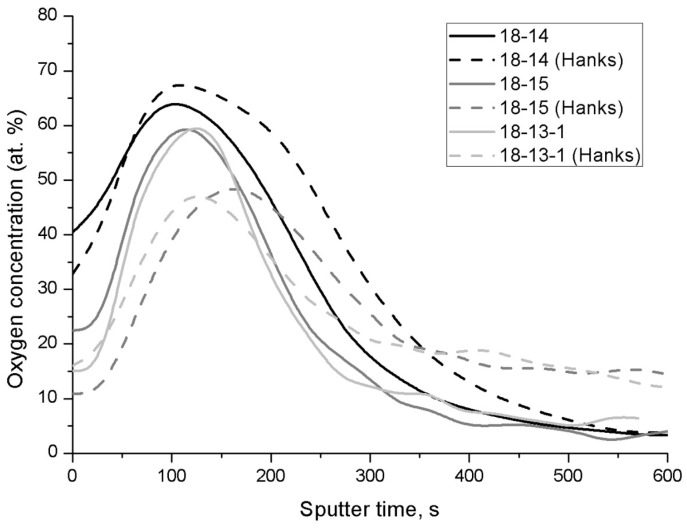
Oxygen concentration depth profiles for the 18-14, 18-15, and 18-13-1 alloys before and after exposure to Hanks’ solution for 604.8 ks.

In order to estimate the oxide layer thickness, the oxygen distribution profiles were obtained for the surface of each sample (Figure 3). The required value can be considered as the inflection point of each curve; it is determined by double differentiation of the “oxygen concentration vs. sputter time” dependence, assuming a sputtering rate of 8.3·10^–2^ nm/s. The results are listed in Table 5.

The results in Table 5 indicate an increase in the oxide film thickness upon exposure to Hanks’ solution, which is related to the additional oxidation in the medium of the samples tested. For the 18-14 and 18-15 samples, the oxide film thickness increased by 4 and 5 nm, respectively. The exception is the 18-13-1 alloy, which does not show a statistically significant oxide thickening.

### 3.4. Electrochemical Characterization in Static Conditions

**Figure 4:**
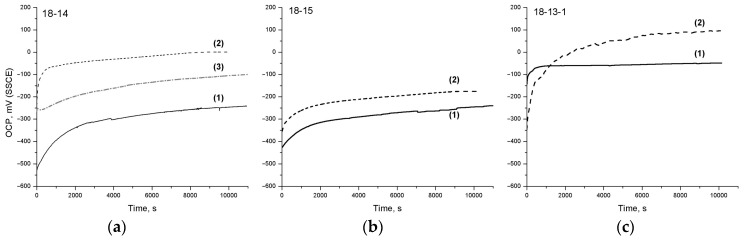
OCP curves of (**a**) 18-14, (**b**) 18-15, and (**c**) 18-13-1 alloys (1) before and (2) after TMT, and (3) the 18-14-based foam material in Hanks’ solution at 310 K.

Figure 4 shows the OCP curves of the studied Ti‒Zr-based alloys before and after TMT, as well as the 18-14-based foam material in Hanks’ solution at 310 K.

The obtained results show that, regardless of the chemical composition and the processing scheme, all the materials exhibit a pronounced anodic polarization effect, which is evidence of the inclination to self-passivation and the formation of protective films of corrosion products (oxides). It can also be seen that TMT leads to a noticeable increase in the OCP steady-state values.

Table 6 summarizes the data concerning the initial (*E_0_*) and steady-state (*E_st_*) OCP values, as well as the resulting OCP shift (*ΔE = E_st_ – E_0_*) during exposure to Hanks’ solution.

**Figure 5:**
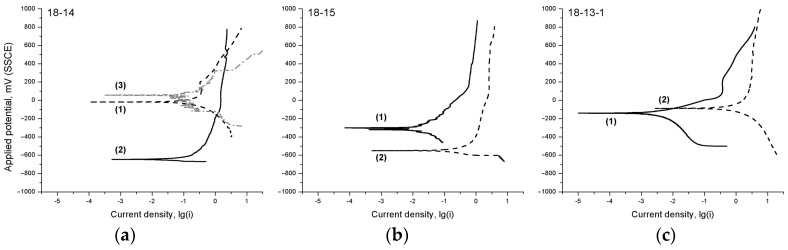
Polarization diagrams of (**a**) 18-14, (**b**) 18-15, and (**c**) 18-13-1 alloys (1) before and (2) after TMT, and (3) the 18-14-based foam material in Hanks’ solution at 310 K.

Figure 5 shows the polarization diagrams of the studied Ti‒Zr-based materials in Hanks’ solution at 310 K.

It can be seen from Figure 5 that, regardless of the chemical composition and the processing scheme, all the materials are in the passive state in a wide range of applied potentials during the anodic polarization. The low anodic dissolution current density (0.4–5 μA/cm^2^) indicates a high corrosion resistance of the studied materials and the nonsusceptibility to pitting corrosion.

A polarization diagram of the 18-14-based foam material features larger current density fluctuations during the potential sweep as compared with the bulk alloy.

### 3.5. Electrochemical Characterization under Cyclic Loading

**Figure 6:**
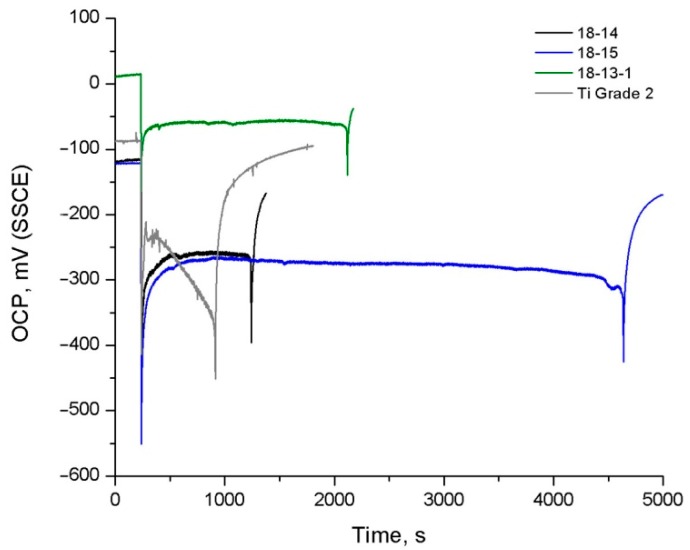
Typical OCP curves of the 18-14, 18-15, and 18-13-1 alloys subjected to TMT and Ti Grade 2 obtained during mechanocycling in Hanks’ solution (1.5% strain, 0.9 Hz).

Cyclic dynamical loading (constant 1.5% strain in bending with 0.9 Hz frequency) of the samples was started after their immersion in Hanks’ solution and establishing the steady-state potential values (*E_st_*) for each alloy. Figure 6 shows the corresponding OCP curves; the main results are given in Table 7, where *E_st_*—the steady-state potential, *E_cs_*—the potential at the moment of cycling start, *E_c_*—the potential established during the cycling, *ΔE*—the corresponding difference between the potential values, and *N_max_*—the number of cycles to failure.

All the curves presented in Figure 6 feature a significant OCP drop at the start, with subsequent partial recovery of the potential values during the ongoing cycling, followed by a second rapid decrease associated with the samples’ fracture. The final OCP growth stage observed for all alloys corresponds to the electrochemical behavior of the freshly fractured surface.

**Figure 7:**
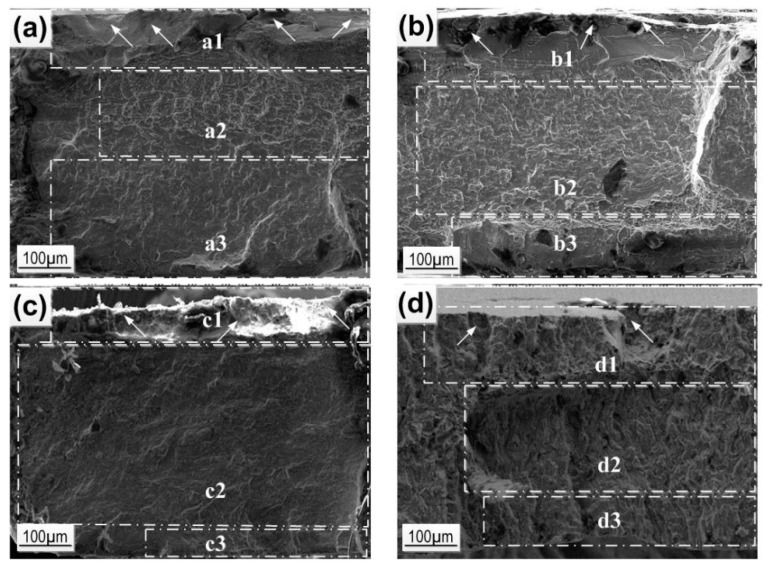
SEM images of (**a**) 18-14, (**b**) 18-13-1, (**c**) 18-15, and (**d**) Ti Grade2 fracture surfaces after mechanocycling in Hanks’ solution: (1) fatigue crack initiation area, (2) the crack propagation area, and (3) mechanical rupture area; arrows indicate the crack initiation points [34].

Figure 7 presents selected SEM images of the fracture surfaces of the 18-14, 18-15 and 18-13-1 alloys and Ti Grade 2 after mechanocycling in Hanks’ solution. In order to analyze the features of the materials’ failure, macroscopically distinguishable areas are marked with white bars in Figure 7: (1) the fatigue crack initiation area, (2) the crack propagation area, and (3) the mechanical rupture area.

## 4. Discussion

### 4.1. XRD Study

XRD results (Figure 1) show that the RT 18-14 and 18-15 alloys consist of β-phase (BCC (body-centered cubic) lattice) only, while the 18-13-1 sample additionally contains α’’-martensite (orthorhombic lattice). Therefore, the martensitic transformation start temperature (*M_s_*) is below RT for 18-14 and 18-15 and above RT for 18-13-1. At the same time, Nb is known to be an effective β-stabilizer in titanium alloys; in particular, the addition of 1 atom % Nb leads to a decrease of *M_s_* by about 40 K [24]. Therefore, the *M_s_* of 18-15 is lower than the *M_s_* of 18-14.

The XRD profiles (Figure 1) were used to calculate the lattice parameters of β-phase and α’’-martensite, which are required to estimate the crystallographic resource of recovery strain (*ε_max_*) for each alloy using the Bagaryatskii scheme [46]; the results are listed in Table 8.

It can be seen from Table 8 that the 18-14, 18-15, and 18-13-1 alloys subjected to TMT possess close *ε_max_* values (5.1–5.5%), which are significantly higher than those of the previously studied Ti-22Nb-6Zr (atom %) alloy (~3%) [14]. Higher *ε_max_* values are expected to result in a prolonged functional fatigue life of these superelastic alloys.

### 4.2. Functional Mechanical Behavior

The superelastic loop shape and its evolution during mechanical cycling allow for an effective estimation of the superelastic behavior parameters of the alloys studied.

It follows from Figure 2 that all the alloys studied exhibit pronounced superelastic behavior during tensile tests at RT. The residual strain in the first cycles is lower for the 18-15 alloy than for the 18-14 and 18-13-1 alloys and accounts for ~0.5%, with its value rapidly decreasing during the ongoing mechanocycling, i.e., relatively prompt enhancement of the superelastic behavior is observed for the 18-15 alloy. This is most probably related to its low *M_s_* temperature (see Section 4.1). Such a low *M_s_* point creates a pre-transition state in β-phase at the testing temperature, which leads to a lowering of the transformation yield stress, thus facilitating reversible martensitic transformation under stress. Consequently, the superelastic behavior is stabilized and the functional fatigue life increases, which is confirmed by the highest number of cycles to failure of the 18-15 alloy (*N_max_* = 3275 ± 542); see Table 2.

### 4.3. Surface Film Characterization

In the framework of AES elemental analysis of the surface oxide films on the 18-14, 18-15, and 18-13-1 alloys before and after exposure to Hanks’ solution for 604.8 ks, attention is drawn to the relative concentration of the most reactive elements (Ti and Zr) in the uppermost layer (up to 3 nm) as well as in the near-surface area (about 100 nm), i.e., before and after ion sputtering. Table 9 shows Zr/Ti concentration ratio values for the studied alloys before and after exposure to Hanks’ solution, as calculated from Table 1, Table 3, and Table 4.

The obtained results show that exposure to Hanks’ solution leads to significant surface enrichment with zirconium, which indicates the predominant oxidation of Zr, the latter being associated with higher oxygen affinity as compared to Ti.

Ion sputtering eliminates surface oxide layers; consequently, at a depth of about 100 nm, Zr/Ti ratio values are similar for all alloys and close to the nominal one. The observed mismatch between the near-surface layer composition and the nominal values may be caused by selective sputtering and slight deviations in the elements’ actual relative sensitivity factors from the standardized values.

The oxide film thickness estimation carried out using AES oxygen distribution profiles (Figure 3, Table 5) reveals that the thinnest film, as well as the lowest oxide film thickening after exposure to Hanks’ solution, can be attributed to the 18-13-1 alloy, while the oxide layers on the 18-14 and 18-15 alloys are thicker and grow measurably in the solution environment.

These observations can be explained by a lower imperfection level of the oxide film on the 18-13-1 alloy, which is achieved by substituting a certain number of defects in the lattices of prevailing ZrO_2_/TiO_2_ oxides by tantalum atoms; the total added, however, is small and cannot be reliably detected by AES. The ionic radius of Ta^5+^ ion is comparable to that of Ti^4+^ и Nb^5+^ ions (0.66, 0.64, and 0.66 Å, respectively), which promotes its incorporation into the growing oxide layer. It is also worth noting that TiO_2_ is an anion-deficient n-type semiconductor. According to the Wagner‒Hauffe theory, the doping of TiO_2_ with higher valence ions leads to a decrease in the defect concentration and corrosion rate reduction [47].

### 4.4. Electrochemical Characterization in Static Conditions

The analysis of the OCP curves obtained during the exposure to Hanks’ solution (Figure 4 and Table 6) shows that the highest steady-state potential values *E_st_* before and after TMT are exhibited by the 18-13-1 alloy, which can be a consequence of its passive film’s advanced protective properties (see Section 4.3). For the 18-14 and 18-15 alloys, the E_st_ values are comparable before TMT, while after TMT, the *E_st_* of the 18-14 alloy is 170 mV higher.

The 18-14-based foam material exhibits a significantly higher OCP than the bulk 18-14 alloy, which can be related to the residual partially oxidized and more developed surface that could not be removed by polishing prior to OCP measurements. This corresponds to a relatively small *ΔE* shift during exposure to the solution.

Generally, a positive effect of TMT on the OCP behavior of the studied alloys is revealed. In particular, steady-state potential values *E_st_* increase; the 18-15 alloy, after TMT, additionally features a larger *ΔE* shift, which indicates an improvement in the surface’s protective ability upon exposure to Hanks’ solution. The initially small *ΔE* value for the 18-13-1 alloy before TMT correlates with the insignificant change in its oxide layer thickness during the exposure (Table 5).

In order to reveal the mechanisms of the passivating film formation and the kinetic regularities of their growth during the exposure to Hanks’ solution, the OCP curves were processed, taking into account the proportional dependence between the OCP values and the passive layer thicknesses. The approach is described in more detail in [48].

For each time moment *t* and corresponding OCP value *E_t_*, the normalized potential change was calculated via Equation (1):

(1) $$e_{t}=\frac{\Delta E_{t}}{\Delta E}=\frac{E_{t}-E_{0}}{E_{\infty }-E_{0}}.$$

The analysis shows that all normalized OCP curves can be approximated with a high confidence level (*R^2^* > 0,99, Table 10) with two logarithmic dependences describing the variations of *e_t_* in two time periods—the initial period (A) and the residual period (B) corresponding to Equations (2) and (3), respectively. The equations are as follows:

A (2) e=a+kln(ct+1)

B (3) $$e=\frac{1}{a-k\ln\left(t+c\right)},$$

where *a*, *k*, *c* are fitting parameters.

The time moment where the A-region changes to the B-region (*t_x_*) was adjusted to obtain the best reliability in OCP curve fitting (highest *R^2^* values) using Origin 8.0 software (OriginLab Corp., Northampton, MA, USA). These regions correspond to different mechanisms that define the growth rate of surface oxide films.

The kinetic regularities corresponding to Equations (2) and (3) were introduced by Cabrera and Mott [49] and Hauffe and Ilschner [50], respectively, to describe the growth of thin oxide films (less than 5 nm) at low temperatures. According to their concerns, the formation of the thinnest layers at the initial stage should follow the direct logarithmic time dependence (A) with a subsequent change to the reciprocal logarithmic one corresponding to different mechanisms that limit the film growth and, therefore, define the process rate—electron tunneling through the thinner oxide film and ion transfer through the thicker oxide film.

The parameter *k* in Equations (2) and (3) can be considered rate constants of oxide film formation. The OCP curves processing results are summarized in Table 10.

It follows from Table 10 that the oxide film growth rate increases significantly after TMT, which is indicated by the decrease in *t_x_* values (the thinnest film formation stage (A) terminates earlier). This trend can be explained by the formation of a more developed surface substructure leading to an increase in surface reactivity and an intensification of the film’s growth.

The highest *t_x_* value is observed for the 18-14-based foam material, which can be related to the hindered inlet of oxygen to some parts of the developed surface, resulting in the inhibited oxide layer growth.

Before TMT, the 18-14 alloy demonstrates the highest inclination to self-passivation, considering the highest *k* value in period (A). It can be noted that the most considerable influence of TMT on the film growth kinetics is evident for the 18-14 alloy—parameter *c* in region (A) in the case of the sample after TMT is two orders of magnitude greater than that of the sample before TMT, while for 18-15 and 18-13-1 the difference is not so sharp.

The polarization diagrams of the studied alloys (Figure 5) feature a change in the cathodic (lower) part of the curve, which is associated with the overvoltage decrease (release) of the rate-controlling cathodic process; in the case of Hanks’ solution, this is oxygen depolarization reaction O_2_ + 2H_2_O + 4ē = 4OH¯.

It is known that the indirect comparative aspect of the electrode process’ inhibition is the slope of the polarization curve. It can be seen that Ti‒Zr-based materials before TMT are characterized by a much steeper slope of the cathodic part of the polarization diagrams compared with after TMT. The reason for this cathodic process release after TMT may be an increase in the amount of orthorhombic face-centered α’’-martensite acting as a cathode, i.e., the surface regions where the cathodic process can take place.

In addition, it is reasonable to assume that, during TMT, the redistribution of interstitial impurities can take place, with their segregation regions being the additional local zones favoring the cathodic reaction. It is also probable that the oxide film forming on the surface after TMT is more heterogeneous than in the case with no TMT [51], with more oxidized surface areas acting as the cathodic regions.

In the case of the 18-14-based foam material, the increase in corrosion rate, as well as the current density fluctuations observed in the polarization curve, may be related to the developed surface of the sample and the local passivation instability caused by the deficiency in oxygen required to form a highly protective oxide film.

### 4.5. Electrochemical Characterization under Cyclic Loading

For all alloys, the cycling start is accompanied by a drastic drop of OCP towards more negative values. This shift is associated with the cathodic polarization resulting from the local passive film destruction.

At the initial stage of cycling, the OCP drop *ΔE* is bigger for the 18-15 samples than for other alloys (Table 7), which indicates higher oxide film damageability under mechanical loading.

It must be noted that the OCP behavior during the cycling of superelastic Ti‒Zr-based alloys and non-superelastic Ti Grade 2 is different. For superelastic alloys, during cycling, an increase in OCP is observed, which is related to the plastifying impact of the chemomechanical effect, which takes place at the sample/electrolyte interface [52,53,54], with the subsequent establishment of a constant OCP value during cycling, *E_c_*. As follows from Table 7, the highest *E_c_* values are observed for the 18-13-1 alloy, which can be explained by the higher permeability of thinner oxide films, which does not facilitate the accumulation of dislocations in the near-surface area.

In the case of Ti Grade 2, during about the first 50 cycles, recovery of the potential values is observed, related to the reconstruction of the damaged oxide layer and chemomechanical effect. Further OCP decrease indicates the impossibility of restoring the passive state during the unloading half-cycle. After about 900 cycles, the sample failure takes place (Table 7).

The highest number of cycles to failure is provided by the 18-15 alloy (*N_max_* = 4225 ± 677) and this gain is caused, to a great extent, by the favorable *M_s_* temperature point resulting in an equally prolonged fatigue life in air (see Section 4.2).

Fracture images analysis (Figure 7) shows that the largest area of the fatigue crack propagation and the smallest mechanical rupture zone are observed for the 18-15 alloy and correspond to its outstanding fatigue life. The delayed failure of this alloy is evidently connected with the effective slowdown of the crack propagation due to the formation of martensite crystals at its tip as well as the stress relaxation via the martensitic transformation mechanisms [53,54].

For the 18-13-1 alloy, the crack propagation area is closer in size to the rupture zone; for the 18-14 alloy, it exceeds it, which corresponds to fewer cycles to failure.

For Ti Grade 2, the crack propagation zone is significant, but due to the absence of a reversible martensitic transformation, it shows the worst functional fatigue behavior in Hanks’ solution.

## 5. Conclusions

In the present study, several prospective Ti‒Zr-based superelastic materials (Ti-18Zr-14Nb, Ti-18Zr-15Nb, Ti-18Zr-13Nb-1Ta, atom %) were produced and characterized.

1. The studied alloys were subjected to thermomechanical treatment (TMT), comprising cold rolling with true strain of *e* = 0.3 + annealing at 873 K, 1.8 ks, consisting only of BCC β-phase at room temperature (RT) in the case of the 18-14 and 18-15 alloys, while the 18-13-1 alloy also contains orthorhombic α”-martensite, which gives the following relation of *M_s_* temperatures: *M_s_* (18-15) < *M_s_* (18-14) < RT < *M_s_* (18-13-1).
2. The crystallographic limits of recovery strain calculated for the 18-14, 18-15, and 18-13-1 alloys subjected to TMT amount to 5.1–5.5%, which are significantly higher than those of the previously studied Ti-22Nb-6Zr (atom %) alloy (~3%).
3. By means of Auger electron spectroscopy, in combination with ion sputtering, it is shown that before and after exposure to Hanks’ solution for 604.8 ks, the alloys’ surfaces are substantially enriched with zirconium. The oxide layer on the 18-13-1 alloy possesses the lowest thickness, which is probably related to its low imperfection level, which is additionally confirmed by an insignificant increase in the oxide film thickness during exposure to Hanks’ solution.
4. By means of open-circuit potential and polarization diagram measurements in Hanks’ solution at 310 K, it is shown that the 18-14, 18-15, and 18-13-1 alloys before and after TMT, as well as the 18-14-based foam material, all exhibit a distinct tendency to self-passivation behavior under these conditions. TMT intensifies this phenomenon. The kinetics of surface oxidation of the 18-14-based foam material is mainly described by the direct logarithmic dependence, which indicates the formation of thinner oxide films. It should be noted that the revealed differences in the foam material electrochemical behavior compared with bulk state do not hinder its application as a corrosion-resistant material in the human body.
5. Based on the results of multi-cycle functional fatigue testing (tensile straining to 2% with subsequent unloading) as well as of mechanical cycling in Hanks’ solution (bending strain of 1.5% and a frequency of 0.9 Hz), it is shown that the highest number of cycles to failure is exhibited by the 18-15 alloy, which is mostly related to the more complete realization of the reversible martensitic transformation upon loading, making it more prospective material for load-bearing implants than the other alloys studied

## Figures and Tables

**Table 1:** Chemical composition of the Ti‒Zr alloys, in atom %.

Designation	Ti	Zr	Nb	Ta
18-14	68	18	14	–
18-15	67	18	15	–
18-13-1	68	18	13	1

**Table 2:** The average number of cycles to failure (*N_max_*) during 2%-strain cycling of the 18-14, 18-15, and 18-13-1 alloys subjected to TMT.

Alloy	*N_max_*
18-14	826 ± 198
18-15	3275 ± 542
18-13-1	1796 ± 440

**Table 3:** Surface elemental compositions of the 18-14, 18-15, and 18-13-1 alloys at different analysis depths (atom %); before exposure to Hanks’ solution.

Element	18-14		18-15		18-13-1	
	3 nm	100 nm	3 nm	100 nm	3 nm	100 nm
O	85	20	87	15	80	14
Ti	10	49	7	54	11	54
Zr	2	17	5	17	2	17
Nb	2	14	1	14	7	14
Ta	–	–	–	–	–	–

**Table 4:** Surface elemental compositions of the 18-14, 18-15, and 18-13-1 alloys at different analysis depths (atom %); after exposure to Hanks’ solution.

Element	18-14		18-15		18-13-1	
	3 nm	100 nm	3 nm	100 nm	3 nm	100 nm
O	44	7	48	11	46	14
Ti	6	54	2	46	6	50
Zr	5	17	4	16	4	14
Nb	5	14	4	14	3	14
Ta	–	–	–	–	–	–
Ca	19	2	17	3	17	5
Na	18	5	20	8	15	6
P	2	1	3	1	3	–
Cl	1	–	2	1	3	1
Total (of solution components)	40	8	42	13	38	12

**Table 5:** Oxide film thicknesses estimated for the 18-14, 18-15, and 18-13-1 alloys before and after exposure to Hanks’ solution.

Alloy	Oxide Film Thickness, h ± 2 nm	
	Before Exposure	After Exposure
18-14	18	22
18-15	16	21
18-13-1	14	15

**Table 6:** OCP curve features for the 18-14, 18-15, and 18-13-1 alloys before and after TMT, and the 18-14-based foam material in Hanks’ solution at 310 K.

Alloy	TMT	*E*, mV (SSCE)		
		*E_0_*	*E_st_*	*ΔE*
18-14	Before	−529	−241	288
	After	−241	1	240
18-15	Before	−427	−244	183
	After	−355	−175	180
18-13-1	Before	−131	−48	83
	After	−346	99	445
18-14 (foam)	−	−265	−96	169

**Table 7:** Electrochemical parameters and functional fatigue life (*N_max_*) of the 18-14, 18-15, and 18-13-1 alloys and Ti Grade 2 obtained during mechanocycling in Hanks’ solution.

Alloy	*E_st_*	*E_cs_*	*ΔE_(Ecs-Est)_*	*E_c_*	*N_max_*
18-14	−118	−514	−396	−258	1054 ± 328
18-15	−76	−588	−512	−270	4225 ± 677
18-13-1	14	−165	−179	−56	1806 ± 559
Ti Grade 2	−87	−413	−326	(−235)	898 ± 318

**Table 8:** Lattice parameters and crystallographic resource of recovery strain (*ε_max_*) of the 18-14, 18-15, and 18-13-1 alloys.

Alloy	β-phase				α”-martensite			*ε_max_*, %
	*a*, Å			*a*_extr,_ Å	*a*, Å	*b*, Å	*c*, Å	
	110	200	211		200	020	002	
18-14	3.347	3.338	3.336	3.330 ± 0.002	3.174 ± 0.005	4.982 ± 0.011	4.968 ± 0.015	5.2 ± 0.2
18-15	3.354	3.334	3.338	3.337 ± 0.005	3.191 ± 0.008	4.986 ± 0.013	4.813 ± 0.016	5.1 ± 0.2
18-13-1	3.348	3.347	3.347	3.346 ± 0.001	3.187 ± 0.004	4.996 ± 0.009	4.852 ± 0.011	5.5 ± 0.2

**Table 9:** Concentration ratio Zr/Ti for the 18-14, 18-15, and 18-13-1 alloys derived from the AES results obtained from the uppermost layer (1) and the near-surface layer (2), before and after exposure to Hanks’ solution for 604.8 ks; the last column provides nominal Zr/Ti concentration ratio values for each alloy.

Alloy	Before Exposure		After Exposure		Nominal Value
	Zr/Ti (1)	Zr/Ti (2)	Zr/Ti (1)	Zr/Ti (2)	
18-14	0.20	0.35	0.83	0.31	0.26
18-15	0.71	0.31	2.00	0.35	0.27
18-13-1	0.18	0.31	0.67	0.28	0.26

**Table 10:** The results of OCP curves approximation for the 18-14, 18-15, and 18-13-1 alloys before and after TMT, and the 18-14-based foam material in Hanks’ solution at 310 K.

Alloy	TMT	Period	*t_x_, s*	*a*	*k*	*c*	*R^2^*
18-14	Before	A	2600	−0.002	0.370	0.003	0.999
		B		2.882	0.206	−1086	0.994
	After	A	700	−0.064	0.156	0.237	0.998
		B		2.831	0199	723	0.996
18-15	Before	A	2900	−0.002	0.232	0.009	0.999
		B		4.030	0.330	586	0.998
	After	A	1800	−0.012	0.383	0.002	0.999
		B		3.901	0.313	−706	0.990
18-13-1	Before	A	800	−0.067	0.225	0.068	0.989
		B		1.281	0.017	−750	0.962
	After	A	600	0.010	0.267	0.010	0.991
		B		2.843	0.207	−587	0.986
18-14 (foam)	−	A	5500	−0.012	0.557	0.001	0.996
		B		3.373	0.262	−3217	0.999
